# An exploration of the ability of tepoxalin to ameliorate the degradation of articular cartilage in a canine *in vitro *model

**DOI:** 10.1186/1746-6148-5-25

**Published:** 2009-07-22

**Authors:** Lisa Macrory, Anne Vaughan-Thomas, Peter D Clegg, John F Innes

**Affiliations:** 1 Musculoskeletal Research Group, Faculty of Veterinary Science, University of Liverpool, Leahurst Campus, Neston, Wirral, CH64 7TE, UK

## Abstract

**Background:**

To study the ability of tepoxalin, a dual inhibitor of cyclooxygenase (COX) and lipoxygenase (LOX) and its active metabolite to reduce the catabolic response of cartilage to cytokine stimulation in an *in vitro *model of canine osteoarthritis (OA).

Grossly normal cartilage was collected post-mortem from seven dogs that had no evidence of joint disease. Cartilage explants were cultured in media containing the recombinant canine interleukin-1*β *(IL-1*β*) at 100 ng/ml and recombinant human oncostatin-M (OSM) at 50 ng/ml. The effects of tepoxalin and its metabolite were studied at three concentrations (1 × 10^-5^, 1 × 10^-6 ^and 1 × 10^-7 ^M). Total glycosaminoglycan (GAG) and collagen (hydroxyproline) release from cartilage explants were used as outcome measures of proteoglycan and collagen depletion respectively. PGE_2 _and LTB_4 _assays were performed to study the effects of the drug on COX and LOX activity.

**Results:**

Treatment with IL-1*β *and OSM significantly upregulated both collagen (p = 0.004) and proteoglycan (p = 0.001) release from the explants. Tepoxalin at 10^-5 ^M and 10^-6 ^M caused a decrease in collagen release from the explants (p = 0.047 and p = 0.075). Drug treatment showed no effect on GAG release. PGE_2 _concentration in culture media at day 7 was significantly increased by IL-1*β *and OSM and treatment with both tepoxalin and its metabolite showed a trend towards dose-dependent reduction of PGE_2 _production. LTB_4 _concentrations were too low to be quantified. Cytotoxicity assays suggested that neither tepoxalin nor its metabolite had a toxic effect on the cartilage chondrocytes at the concentrations and used in this study.

**Conclusion:**

This study provides evidence that tepoxalin exerts inhibition of COX and can reduce *in vitro *collagen loss from canine cartilage explants at a concentration of 10^-5 ^M. We can conclude that, in this model, tepoxalin can partially inhibit the development of cartilage degeneration when it is available locally to the tissue.

## Background

Osteoarthritis (OA) is the most common arthropathy of mammals and affects approximately 20% of the adult canine population [1]. The condition is typically treated with nonsteroidal anti-inflammatory drugs (NSAIDs), often for many months or years.

OA is characterised by the degeneration and loss of articular cartilage. The single cell type of cartilage is the chondrocyte and this cell is the architect of an extracellular matrix consisting mainly of water, type II collagen and proteoglycans [2]. The major proteoglycan of cartilage, aggrecan, contains large numbers of glycosaminoglycan (GAG) side-chains. Collagen provides the tensile strength to the tissue whereas aggrecan imbibes water thereby providing compressive stiffness [3]. In canine OA, an early event is the loss of aggrecan through degradative enzymic activity by aggrecanases (ADAMTS-4, -5) [4]. Aggrecan loss renders the collagen network susceptible to degradation by the matrix metalloproteases, MMP-1 and -13 [5,6]. The overall effect of increased degradation and inappropriate synthesis is a gradual loss of tissue. It is thought that the upregulation of matrix degradation and downregulation of synthesis is mediated through catabolic cytokines such as IL-1β, IL-6 and oncostatin-M (OSM) [7-9].

Culturing cartilage explants is a well established tool for studying inflammatory processes and *in vitro *models of cartilage degradation in several domestic species have been developed [10,11] including species of veterinary interest [12-14]. These models utilize a variety of catabolic cytokines to induce cartilage degradation and depress matrix synthesis in explants of cartilage retrieved from normal joints. The release of these organic components from the explants can be measured by using assays for glycosaminoglycan and hydroxyproline (a direct measure of collagen release) on the culture media.

There is clearly great interest in drugs that might retard, stop or reverse the biological processes of OA and preserve cartilage tissue integrity. Because NSAIDs are used routinely in the management of OA pain, often for protracted periods of time, the effect of NSAIDs on cartilage has been previously studied. Early studies in the 1980s demonstrated that in a canine *in vivo *model of OA, aspirin had negative effects on cartilage metabolism [15]. Highlighting the increase in clinical use of agents that show enhanced COX-2 selectivity more recent *in vitro *studies have demonstrated some limited positive effects for carprofen (Rimadyl) on the synthesis of aggrecan [14]. To date, effects on the degradative pathways have received little attention.

Recent work on dual COX-LOX inhibitors has highlighted possible desirable effects on inhibiting both cyclooxygenase and lipoxygenase pathways on cartilage metabolism. As well as the expected inhibition of PGE_2 _and LTB_4 _production, studies of licofelone (ML-3000) in an experimental canine model (Pond-Nuki) of OA demonstrated an ability to decrease progression of OA compared to untreated controls [16]. In particular, this study suggested a decrease in MMP-1 and IL-1β in cartilage in treated groups. Although, a direct comparison was not performed, the investigators suggested that the effect of licofelone was more pronounced than the effects the same investigators had reported with carprofen in a similar model [17]. Furthermore, licofelone has been shown to reduce gene expression of MMP-13, ADAMTS-4 and cathepsin K with corresponding reductions in appearance of protein [18].

Tepoxalin is a dual COX/LOX inhibitor. Previous work has shown that tepoxalin can reduce the production of IL-1 by human synovial tissue explants in organ culture [19], suggesting that tepoxalin may have beneficial effects on cartilage in OA, limiting both the production of endogenous IL-1 and consequent MMP-mediated tissue degradation.

The aim of this study was to use an *in vitro *model of canine OA to investigate the hypothesis that tepoxalin, a dual COX/LOX inhibitor, is able to reduce the catabolic response of cartilage to cytokine stimulation. OSM and IL-1*β *have previously been described as a potent combination in promoting cartilage release in cartilage [7,20]. Total GAG and collagen (hydroxyproline) release in to the tissue culture media were used as outcome measures of proteoglycan and collagen depletion respectively. We demonstrate that tepoxalin, at 10-5 M and 10-6 M, caused a decrease in collagen release from canine articular cartilage explants *in vitro*.

## Methods

### Tissue collection and explant culture

Grossly normal articular load-bearing cartilage was harvested from the femoral condyles and trochlear groove of distal femurs of seven dogs euthanatized for reasons unrelated to musculoskeletal disorders and for reasons unrelated to this study and which had no evidence of gross joint disease on post-mortem examination. Full, informed consent from owners was obtained prior to tissue harvest. Using aseptic technique throughout, the cartilage explants were washed three times in serum-free Dulbecco's Modified Eagle's Medium (DMEM) supplemented with 300 units/ml penicillin and 100 units/ml streptomycin (all from Cambrex, Wokingham, UK).

### Cartilage degradation assay

Individual cartilage explants (approximately 0.75 cm^2^) were placed in 24-well tissue culture plates containing 1 ml of serum-free DMEM supplemented with 100 units/ml penicillin and 100 units/ml streptomycin as described in other studies [21]. Explants in triplicate wells were exposed to treatments with each horse treated as a separate experiment. Recombinant canine (rc) IL-1*β *(R&D Systems, Abingdon, UK) and recombinant human (rh) OSM (Peprotech, London, UK) were added at final concentrations of 100 ng/ml and 50 ng/ml, respectively, to all explant cultures, with the exception of a triplicate negative control group. The effects of tepoxalin and its metabolite were studied at three concentrations (1 × 10^-5^, 1 × 10^-6 ^and 1 × 10^-7 ^M), which have previously been reported to be representative of doses expected to be reached in the human joint during therapy [22]. Additionally, pharmacokinetic data from the technical monograph for the licensed form of tepoxalin (Zubrin, Intervet-Schering Plough Animal Health) and its active metabolite indicate that following the recommended loading dose of 20 mg/kg followed by six days of 10. mg/kg plasma C_max _values range from 1.48 × 10^-6 ^M and 2.8 × 10^-6 ^M suggesting that our selected concentrations spanned the concentrations likely to be achieved in the synovial joint. Cultures were maintained for 28 days (at 37°C in a humidified 5% CO_2_:95% air mixture) and the medium containing stimuli and drug were harvested and replenished every 7 days throughout this period. Harvested media was stored at -20°C prior to GAG and hydroxyproline analysis. Media samples collected at day 7 were assayed for GAG release while collagen degradation and release was measured in media collected at days 7, 14, 21 and 28, all were stored at -20°C prior to assay. To determine the total GAG and collagen content of the explants, the remaining cartilage was digested overnight in 10 units/ml papain (Sigma, UK), 0.1 M sodium acetate, 2.4 mM ethylenediaminetetraecetic acid, 5 mM L-cysteine, pH 5.8 at 60°C.

### Proteoglycan degradation

Media collected on day 7 were assayed for total GAG using the a modification of the dimethylmethylene blue direct dye binding assay [23]. GAG concentration was measured via absorbance at 570 nm with 1,9-dimethylmethylene blue (DMMB) using shark chondroitin sulphate (both Sigma) to establish a standard curve over 5 to 40 μg/ml. Absorbance values were measured using a Dynex revelation 4.04 plate reader (DYNEX technologies) Values were quoted as mg per g wet weight of tissue.

### Collagen degradation

Culture media collected on days 7, 14, 21 and 28 and papain digested tissue were hydrolysed overnight in 6 M HCl at 110°C and subsequently freeze-dried. The lyophilised material was redissolved in distilled water. 40 μl aliquots of redissolved samples or hydroxyproline standards were assayed for total collagen using the hydroxyproline assay as described previously [21].

Absorbance at 560 nm was measured using a Multiskan EX reader (Thermo Scientific) and accumulative amounts of hydroxyproline released over the 28 day culture period into the medium as collagen were calculated as % of total hydroxyproline in the explant.

### Effect of tepoxalin on COX and LOX activity in cartilage

PGE_2 _and LTB_4 _concentrations in media (harvested on day seven) were determined using commercially available competitive ELISA kits (R&D systems), in accordance with the manufacturer's instructions. Plates were read using a Dynex revelation 4.04 plate reader, with absorbance set at 450 nm and corrected by measurements taken at 570 nm. Best fit polynomial standard curves were developed for each plate, and these equations were used to calculate PGE_2 _or LTB_4 _concentrations for samples from each plate.

### Toxicity assays

Grossly normal cartilage was harvested as above from two dogs, with no evidence of joint disease. Explants were cultured in triplicate in 96 well plate format for 24 and 48 hours in 100 μl serum-free media containing catabolic cytokines (100 ng/ml IL-1β and 50 ng/ml OSM) and either tepoxalin or its metabolite at concentrations of 1 × 10^-5 ^M, 1 × 10^-6 ^M or 1 × 10^-7 ^M. Explants were also cultured, in triplicate, in negative (serum free media) and positive control (serum free media containing IL-1*β *and OSM) conditions. Media was harvested at both time points and replaced with 100 μl serum-free DMEM and 10 μl lysis buffer (supplied with kit). Explants were then frozen at -70°C for a minimum of 1 h prior to analysis. Analysis of both the harvested media and the cell lysates liberated from explants were performed using the commercially available Cytox96^® ^non-radioactive cytotoxicity assay kit (Promega, Southampton, UK) which detects release of the enzyme, lactate dehydrogenase, indicating a loss of cellular integrity.

### Statistical analysis

Statistical analysis compared triple replicates of tepoxalin- or metabolite-treated explants from seven individuals to the same number of explants treated with cytokines alone using linear mixed effects regression models, with the donor being the random effect. Where appropriate, the data was transformed to achieve a normal distribution

## Results

### Proteoglycan release from cytokine-treated cultured explants

**Figure 1:**
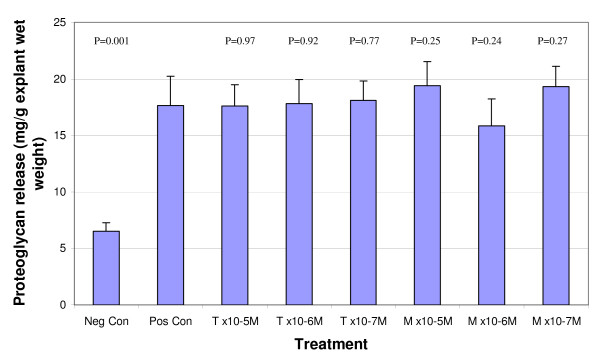
**Effect of tepoxalin and its metabolite on proteoglycan degradation expressed as an average value from four dogs after 7 days of culture**. Explants were cultured in serum free media containing IL-1*β *(100 ng/ml) and OSM (50 ng/ml) along with tepoxalin (T) or its metabolite (M) at ×10^-5^, ×10^-6 ^or ×10^-7 ^M concentrations. Proteoglycan release (GAG in medium) is expressed as milligrams of GAG per gram of wet weight of cartilage. Data represents means + standard error, n = 7. P values correspond to comparisons to the positive control.

Our previous studies of cultured cartilage explants have shown that treatment with IL-1*β *and OSM significantly increase proteoglycan degradation after seven days in culture compared with controls [13]. We used this information to determine whether the addition of tepoxalin or it metabolite would influence the amount of proteoglycan released into the culture media. As shown previously, treatment with IL-1*β *and OSM significantly upregulated proteoglycan degradation by a mean value of 2.7-fold (n = 7, p = 0.001). Treatment with tepoxalin or its metabolite over the concentrations ranges used showed no significant effect on the amount of proteoglycan released from the explants (figure 1).

### Collagen release from cytokine-treated cultured explants

**Figure 2:**
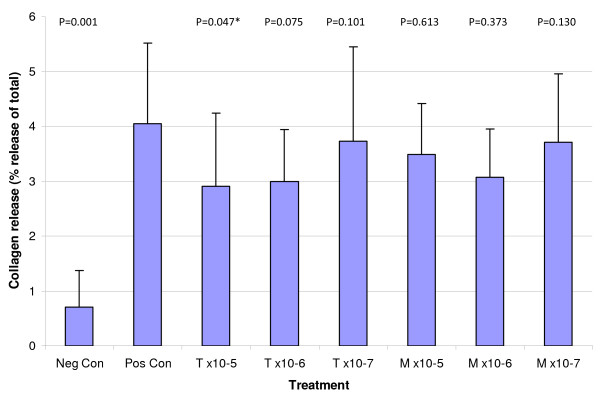
**Average collagen released from explants after 28 days of culture**. Explants were cultured in serum free media containing IL-1β (100 ng/ml) and OSM (50 ng/ml) along with tepoxalin (T) or its metabolite (M) at ×10^-5^, ×10^-6 ^or ×10^-7 ^M concentrations. Values represent means + standard error, n = 7. P values correspond to comparisons to the positive control; * significant at p ≤ 0.05.

In agreement with previous studies, a significant 6.4-fold (mean) increase (p = 0.004) in collagen release (over 28 days) from explants cultured with IL-1*β *and OSM was observed in all dogs with the exception of one donor that was included in the data shown. Treatment with tepoxalin at a concentration of 1 × 10^-5 ^M caused a significant 1.39-fold mean decrease in collagen release (p = 0.047), while treatment with tepoxalin at 10^-6 ^M showed a trend towards significantly reducing collagen release from the explant (a 1.35-fold decrease, p = 0.075) (figure 2). Treatments with tepoxalin at 1 × 10^-7 ^M and its active metabolite caused a minor reduction in collagen released from the explants, however, the effect of these treatments were not statistically significant.

### PGE2 concentrations in media

**Figure 3:**
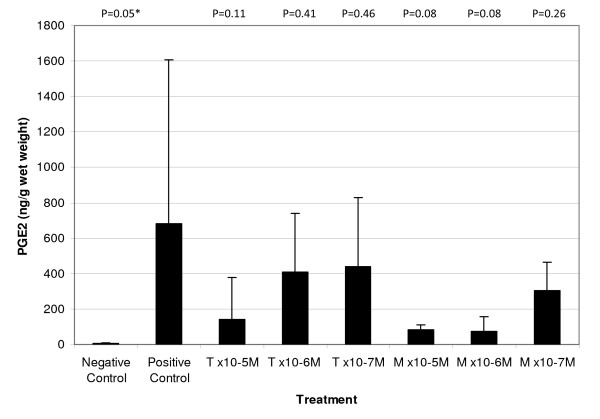
**Effect of tepoxalin and its metabolite on PGE_2 _levels released into culture media of canine cartilage explants**. Media was harvested after seven days of treatment with tepoxalin (T) or its active metabolite (M) at concentrations of ×10^-5^, ×10^-6 ^or ×10^-7 ^M. Values are shown as the mean and SDs from a single donor. P values correspond to comparisons to the positive control; * significant at p ≤ 0.05.

Treatment with OSM and IL-1*β *caused a significant 98-fold increase of PGE_2 _release into the media from explants in the positive control group when compared to media from the negative controls. Treatment with tepoxalin at 1 × 10^-5 ^M, 1 × 10^-6 ^M or 1 × 10^-7 ^M concentrations reduced this level by 4.80-fold, 1.66-fold and 1.55-fold, respectively (figure 3). Treatment with the active metabolite at concentrations of 1 × 10^-5 ^M, 1 × 10^-6 ^M or 1 × 10^-7 ^M also caused a large reduction in the levels of PGE_2 _released from the explants (8.26-fold, 9.22-fold and 2.25-fold, respectively). Concentrations of LTB_4 _in media harvested from explants on day seven were not able to be quantified due to the low levels present (data not shown). Monolayer cultures that were performed for 24 h in an attempt to obtain quantifiable levels of LTB_4 _also failed to achieve levels of LTB_4_, which could be quantified using available assays (data not shown).

### Cytotoxicity of drug treatment

**Figure 4:**
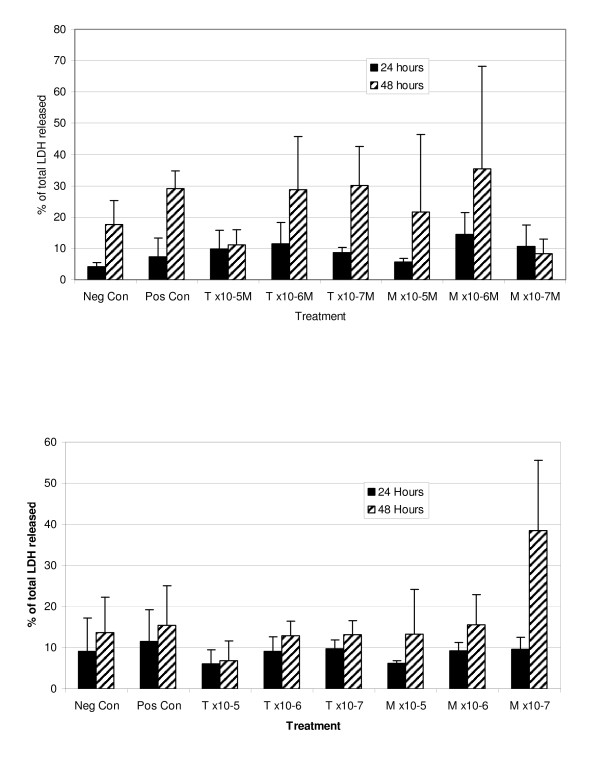
**Percentage of total LDH released from explants when cultured for 24 hours and 48 hours in each drug treatment condition for A) donor 1; B) donor 2**. Explants were cultured in serum free media containing IL-1*β *(100 ng/ml) and OSM (50 ng/ml) along with tepoxalin (T) or its metabolite (M) at ×10^-5^, ×10^-6 ^or ×10^-7 ^M concentrations. Values represent the mean + SDs. There were no significant differences.

Release of the enzyme lactate dehydrogenase (LDH) from cells or explants into the tissue culture medium is a marker for disruption of the cell membrane integrity and an index of cell death. No significant increase in the LDH activity over that measured in explants treated with cytokines alone was observed in the explant culture medium after 24 h treatment nor 48 h treatment with tepoxalin or its metabolite (figure 4).

## Discussion

This study provides evidence that tepoxalin exerts an inhibitory effect on cytokine-induced COX activity and can also, to a certain extent, reduce *in vitro *collagen loss from canine cartilage explants at a concentration of 10^-5 ^M. An increased understanding of the pathophysiology of OA has prompted the development of many drugs and compounds for the treatment of the disease. A number of other drugs with activity directed at inhibiting COX enzymes, such as the NSAIDs, have been in use for many years. Although they very effectively reduce the symptoms of the disease (Husni *et al*., 2001), they have shown limited ability to decrease the *in vivo *progression of experimental OA [17] and may even have adverse direct effects on cartilage [15,24]. More recently, several studies have reported findings indicating that dual COX/LOX inhibitors have enhanced efficacy in delaying progression of the disease. One study [16] reported that ML-3000, a dual COX/LOX inhibitor was more effective in reducing development of OA in a canine model than other NSAIDs, such as tiaprofenic acid and carprofen. It was suggested that the ability of ML-3000 to achieve more effective inhibition of OA progression was due to its ability to inhibit LTB_4 _in addition to PGE_2_.

In order to simulate the progressive degradation of cartilage observed in OA, *in vitro *models using explants of cartilage harvested from the articular surfaces of joints have been developed. In such models, the cartilage is in isolation and the direct effects of biological stimuli or pharmacological agents applied to the cartilage can be investigated. Stimulation of chondrocytes with IL-1*β *results in the inhibition of synthesis of ECM macromolecules such as aggrecan and collagen and also, induces excess production of matrix degrading activities such as MMPs. In agreement with this, our findings showed that treating explants with rcIL-1*β *and rhOSM promotes significant increases in collagen and GAG release. In this study, both tepoxalin and its active metabolite were added to cartilage incubations in order to investigate their activities in the cartilage model. Tepoxalin was found to be able to reduce the IL-1*β*- and OSM-stimulated collagen release but it had no significant effect on GAG release. Collagen degradation requires the combined use of IL-1 and OSM to induce degradation of a relatively small proportion of the total collagen and therefore, the pathways involved may be more amenable to inhibition in this instance. Caution should be exercised before excluding the possibility that its metabolite can also prevent collagen loss *in vivo*, as within the joint, the cartilage is not in isolation and the activity of both tepoxalin and the metabolite on the surrounding tissues may be of importance in preventing the synthesis of inflammatory agents.

We did not observe a dose-dependent effect on collagen release, although our concentrations of tepoxalin and its metabolite were limited to those around the therapeutic range. It is possible that higher concentrations may have had a more dramatic effect but we wanted to only investigate concentrations around the therapeutic range. Clearly higher concentrations would not be possible *in vivo *due to systemic toxicity and potential adverse events. A reduction in collagen degradation is theoretically a beneficial effect [25,26] and, even if this effect is small, because NSAIDs may be used for many months or years, small effects can be important in the development of a slowly-progressive disease such as OA. However, further *in vivo *work would be required to confirm the translation of this initial *in vitro *finding in to an animal model or clinical disease.

The failure to observe an effect on GAG release is perhaps surprising, although it is possible that experimental conditions affected these data. Negative control tissue also released GAG in considerable amounts and this is expected in such experiment, However, it does limit the sensitivity of the system to detect differences between positive and negative control and small effects might go undetected. In addition, the ease of inducing proteoglycan loss in vitro (both IL-1 and OSM will initiate GAG loss whether used in isolation or combination) suggests that the loss may be difficult to inhibit once initiated. That said, previous studies have shown that other NSAIDs such as the COX-2 inhibitor, celecoxib, are able to reduce the release of resident and newly-synthesized GAG in human osteoarthritic cartilage *in vitro *[27]

Our toxicity assay data indicated that neither tepoxalin, nor its active metabolite, had observable toxic effects on chondrocytes in explants at the sampling points of our study. However, although one would expect toxic effects to show in a short timeframe, the sampling points in our study were restricted to 24 and 48 hours of culture.

Both tepoxalin and its metabolite have previously been shown to have a dose-dependent decrease on PGE_2 _levels in an *in vitro *synovial tissue model [28]. Willburger and co-workers [28] reported that the acid metabolite is a pure COX inhibitor on isolated enzymes, and showed that it could inhibit both PG and LTC_4 _release. While the mechanism of action of the metabolite remained uncertain, a direct 5-LO or 12-LO inhibition was excluded. It was therefore hypothesised that other mechanisms such as inhibition of cytosolic phospholipase A_2 _could be a possible route for its activity.

In contrast to the metabolite, tepoxalin can inhibit multiple enzymatic activities (COX, 5-LO, 12-LO) [28]. In this current study, it was found that the metabolite was capable of reducing cytokine-induced PGE_2 _levels to lower levels than corresponding concentrations of tepoxalin, however, unlike tepoxalin it did not significantly reduce collagen loss from the explants. It is therefore possible that the reduced cytokine-stimulated collagen release from explants treated with tepoxalin 10^-5 ^and 10^-6 ^M was a consequence of its inhibition of multiple enzymes and its effect on leukotriene biosynthesis.

Analysis of explants treated with cytokines in the presence of 10^-5 ^M tepoxalin, which is within the therapeutic range, showed both reduced PGE_2 _levels and also decreased level of collagen release from the cartilage explants compared to explants treated with cytokines alone. Further studies are required to investigate the pathways that lead to collagen degradation in order to provide explanation for these findings, however it could be hypothesised that there may be a reduction in the synthesis of IL-1*β *in these explants due to reduced activity of COX, which could in turn be responsible for reduced levels of collagen release. However, while it is well known that PGE_2 _is intimately involved in inflammation, by-products of the 5-LOX pathway, more specifically LTB_4_, have also been shown to play a direct pathogenic role in OA [29]. In the OA synovial membrane, both *in vitro *and *in vivo*, LTB_4 _up-regulates the synthesis of the proinflammatory cytokines IL-1 and tumor necrosis factorα (TNFα) [30] as well as matrix metalloproteases (MMPs).

Our findings show that both tepoxalin, and its metabolite, had a similar trend towards producing a dose-dependent reduction in PGE_2 _levels. Unfortunately, despite using a variety of *in vitro *models, we were unable to obtain quantifiable levels of LTB_4_. However, LTB_4 _(and LTC_4_) have been reported to originate almost exclusively from the synovial tissue, rather than articular cartilage (Wittenberg *et al*., 1993), which may provide an explanation for the low levels of LTB_4 _observed in this study. Consequently in this study we were unable to quantify LTB_4 _due to the limited sensitivity of the assays to the very low levels of ligand available.

## Conclusion

In conclusion, this study demonstrated that tepoxalin, a dual inhibitor of COX/LOX statistically significantly reduced collagen loss in an *in vitro *experimental model of OA. While neither tepoxalin, nor its metabolite, could inhibit GAG loss *in vitro*, tepoxalin alone inhibited loss of collagen. The mechanisms underlying this difference need further investigation.

## Authors' contributions

LM carried out the experiments, completed some of the statistical analyses and drafted the manuscript. AV-T advised on design of laboratory experiments, data interpretation and helped draft the manuscript. PDC participated in the design of the study, advised and partially performed the statistical analysis, and helped draft the manuscript. JI conceived of the study, and participated in its design and coordination, and helped to draft the manuscript. All authors read and approved the final manuscript.
